# Correction to “Impact of Stress Hyperglycemia Ratio on Adverse Outcomes in Patients With Chronic Thromboembolic Pulmonary Hypertension”

**DOI:** 10.1111/cts.70754

**Published:** 2026-11-05

Y. Wang , X. Li , Z. Huang , et al., “Impact of Stress Hyperglycemia Ratio on Adverse Outcomes in Patients With Chronic Thromboembolic Pulmonary Hypertension,” Clinical and Translational Science 18, no. 12 (2025): e70426, 10.1111/cts.70426.PMC1267096441328963

In the article cited above, the funding sources were not listed in the intended order. The Funding section should have read:

“This research article was supported by the National Key Research and Development Program of China (2023YFC2507203); Noncommunicable Chronic Diseases‐National Science and Technology Major Project (2024ZD0538200); Chinese Academy of Medical Sciences Innovation Fund for Medical Sciences (2023‐I2M‐C&T‐B‐063); National High Level Hospital Clinical Research Funding (2022‐GSP‐GG‐35, 2025‐GSP‐QN‐21); The Artificial Intelligence and Information Technology Application Fund of Fuwai Hospital, Chinese Academy of Medical Sciences (2024‐AI26); Beijing Natural Science Foundation (L252080); China Postdoctoral Science Foundation (2024M760273); Postdoctoral Fellowship Program (Grade C) of China Postdoctoral Science Foundation (GZC20251380). The funding source(s) had no involvement in study design or in the collection, analysis or interpretation of data or in the writing of the report or in the decision to submit the article for publication.”

We apologize for this error.

